# A call to action for universal health coverage: Why we need to address gender inequities in the neglected tropical diseases community

**DOI:** 10.1371/journal.pntd.0007786

**Published:** 2020-03-12

**Authors:** Kim Ozano, Laura Dean, Mami Yoshimura, Eleanor MacPherson, Natalia Linou, Mariam Otmani del Barrio, Christine M. Halleux, Olumide Ogundahunsi, Sally Theobald

**Affiliations:** 1 Department of International Public Health, Liverpool School of Tropical Medicine, Liverpool, United Kingdom; 2 United Nations Development Program, New York, United States of America; 3 Malawi-Liverpool-Wellcome Trust Clinical Programme, Blantyre, Malawi; 4 UNICEF/UNDP/World Bank/WHO Special Programme for Research and Training in Tropical Diseases (TDR), World Health Organization, Geneva, Switzerland; UNITED STATES

The UN’s Sustainable Development Goals (SDGs) and pledge to leave no one behind have raised the importance of ensuring equitable health outcomes and healthcare delivery. Multisectoral approaches to tackling neglected tropical diseases (NTDs), including prevention, diagnosis, treatment, and healthcare, have had a limited focus on gender. Yet, gender roles and relations shape vulnerability to NTDs, access to prevention and treatment, and experience of living with NTDs [1]. Understanding the similarities and differences of disease vulnerability and experience between genders can support NTD actors to deliver equitable prevention, diagnosis, and treatment services. The NTD community, including researchers and practitioners, needs to better understand these dynamics and take action to advance gender equality, meet the NTD roadmap 2020 goals, and contribute towards the SDGs and universal health coverage (UHC). The UHC movement is advocating for clear action to address the gender determinants of health. This viewpoint synthesizes evidence from a discussion paper [2] developed by the UN Development Programme (UNDP) and TDR (Special Programme for Research and Training in Tropical Diseases sponsored by UNICEF, UNDP, World Bank, and WHO) in partnership with the Liverpool School of Tropical Medicine to support governments and nongovernment organizations to understand how to recognize and address gender inequities within NTD programs and improve delivery through gender analysis.

Gender refers to the socially constructed roles, behaviors, activities, attributes, and opportunities that any society considers appropriate for men and women, boys and girls, and people with nonbinary identities [3, 4]. Gender, sex, and their intersections with other social determinants of health shape peoples’ vulnerability to and experience of multiple NTDs and their ability to access care and treatment [3, 5]. This can be complex and will vary between diseases, contexts, and other social and demographic factors such as age. Fig 1 highlights differences in disability-adjusted life years (DALYS) from different NTDs by age and sex from the Global Burden of Disease (GBD) Study [6].

**Fig 1:**
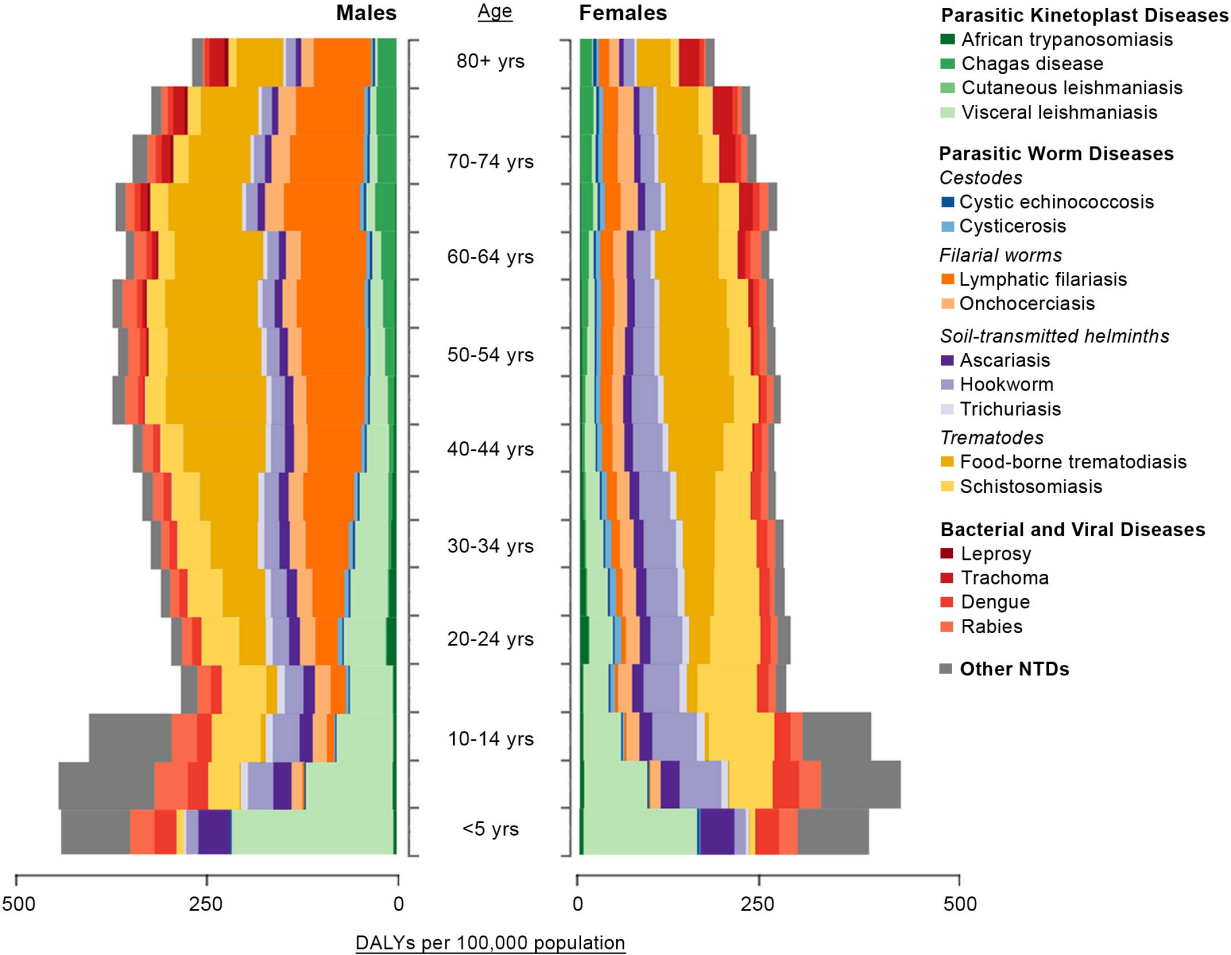
DALYs per 100,000 population by age and gender in 2013 from Herricks and colleagues [6]. DALY, disability-adjusted life year; NTD, neglected tropical diseases.

By examining how gender shapes who is infected with NTDs, who accesses preventive medicines, who is diagnosed and treated, who is exposed or vulnerable to NTDs, and how and whose behavior is risk prone or risk averse, inequities can be better understood, challenged, and addressed.

Fig 2 provides an illustrative example of how sex and gender differentials impact exposure, transmission, manifestation, and treatment for genital schistosomiasis.

**Fig 2:**
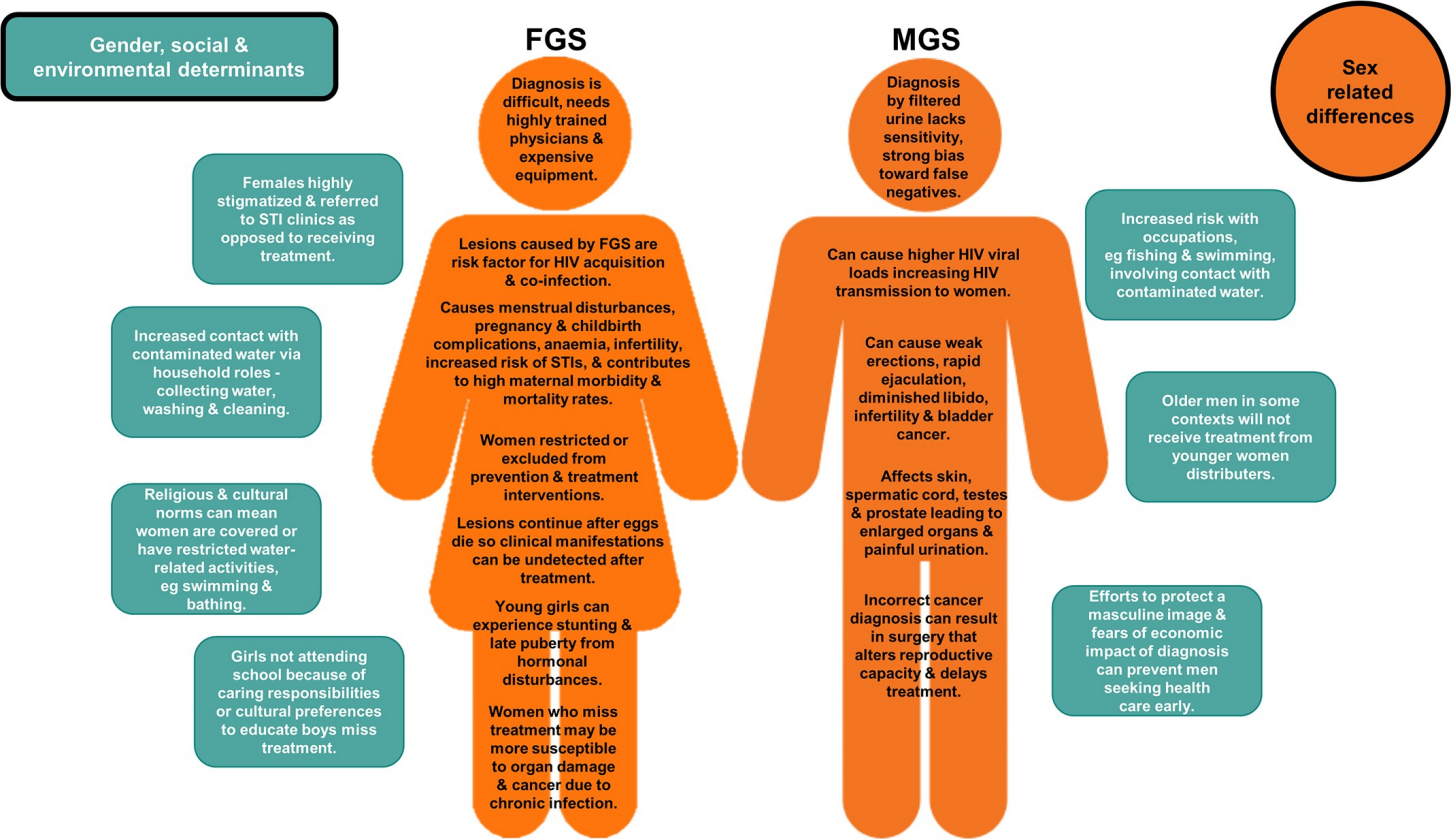
An illustrative example of how sex and gender differentials impact exposure, transmission, manifestation and treatment for genital schistosomiasis [7–11]. FGS, female genital schistosomiasis; MGS, male genital schistosomiasis.

Gender analysis and mainstreaming in policy development, advocacy, legislation, resource allocation, planning, implementation, and monitoring of NTD programs can help the NTD community move beyond dialogue to action [5]. It is also important to consider how rapidly changing environmental and political contexts due to conflict, climate change, urbanization, and migration, intersect with individual characteristics to affect levels of infection for people of different genders and how it impacts their vulnerability to infection, healthcare seeking behavior, and delivery of prevention and treatment programs. This requires a whole-society approach that engages civil society, patients’ rights advocacy groups, communities, private sector (e.g., pharma), UN agencies, and bi/multilateral donors to ensure NTD programs meet the needs of all genders. We propose a set of five recommendations for consideration.

## Recommendation 1: Account for how gender-related division of labor, everyday practices, social norms, and beliefs within and beyond the household impact NTD risk

The division of labor within and beyond the household and everyday practices intersects with other determinants such as age and socioeconomic status to affect exposure to NTD risks. For example, in contexts such as the Republic of Congo (where fishing, farming, hunting are the main income activities), prevalence of lymphatic filariasis (LF) in men is higher, especially where men sleep outside during these activities [12]. However, where people have similar occupational activities, such as agricultural work (as found in rural India), LF infection patterns among men and women were almost equal [13]. Understanding who does what in paid and unpaid labor and in the everyday practices of people of different genders in relation to demographics and social determinants and their impact on NTD experience is important when designing effective health promotion campaigns, e.g., developing protective factors such as bed nets and vector control mechanisms and identifying hot spots for NTDs [13]. As shown in Fig 2, women, men, boys, and girls are exposed differently to urogenital schistosomiasis based on variation of reproductive and productive activities. Such activities are influenced by the broader social context and should be considered to enable context specific responses.

## Recommendation 2: Account for how gender impacts the accessibility and acceptability of treatment

### Preventive chemotherapy

Gender relations, occupation, and other social factors affect accessibility and acceptability of medicines delivered by community drug distributers (CDDs) during mass drug administration (MDA) [9]. Gender disaggregated data collection that also reflects the gender of the distributer will help shed light on potential gendered biases in MDA delivery. The gender of CDDs matters and programs should be supported with a set of probing questions to help district- and local-level implementers consider who is recruited as a CDD and where they will be best placed (Box 1) [5]. Providing training and supportive supervision structures would also help CDDs reflect on how they promote gender equity in their work and to consider which coverage improvement strategies will be most appropriate in different contexts [5].

**Box 1. Questions to consider when recruiting CDDs, adapted from Theobald and colleagues**

Critical questions to help district and local level implementers consider who is recruited as a CDD, how this is influenced by broader power relations, and where the CDDs will be best placed.

- Who is chosen to distribute the drugs and why? (How is this shaped by community members ability to participate?)
- How are they chosen, and who is involved? (What institutions or individuals are making the selection?)
- Does the CDD’s gender affect their ability to access certain household members or enter the home? (What is their access to specific resources or social networks?)
- Does this access also influence individual, household, and community adherence?
- What are the coverage improvement strategies?
- Who decides on them?

### Intensified case management, health seeking, diagnosis and holistic treatment

Intensified case management (ICM) for NTDs not treatable through preventive chemotherapy involves ongoing care for affected individuals and those at risk of infection. The interplay between poverty, gender, age, disability, and other axes of inequity can affect an individual’s decision to access healthcare or outreach services for screening, diagnosis, and case management [14]. For example, studies in India have demonstrated that women with signs of NTDs often delayed seeking healthcare until their husband or guardian agreed and, as a result, had more severe disease outcomes [3, 15]. For men, hegemonic masculinity and prevailing gendered norms, which combine sexual functioning and social responsibilities, can present barriers to accessing diagnosis and ongoing treatment, as found in Ghana [16].

To address inequities found within ICM of NTDs, education of frontline health workers (including CDDs) is necessary to provide accurate information through better communication techniques. National programs that offer culturally acceptable health education to promote early reporting of disease signs could support early access at health services [15]. The use of participatory approaches to understand the ongoing needs and priorities of affected individuals (not just those already engaged with the health system) is also essential to the development of more equitable person-centered health systems. Such approaches can explore variations in experience based on gender and other intersecting axes of inequity and, through the promotion of community-based collective action, can support in the establishment of gender-transformative processes that are led by communities.

## Recommendation 3: Address gender-related stigma and mental health impacts of NTDs

Understanding NTD-related stigma and how it is affected by gender norms will help to minimize the negative impact of stigma, reduce discrimination, support social acceptance, improve disease control and knowledge, and prevent disability [17]. Constructions of mental health often vary significantly across contexts and between genders [18]. For example, women suffering from onchocerciasis, leprosy, and LF describe feelings of embarrassment, shame, sadness, despair, and fear in studies from the Dominican Republic, West Africa, and India [15, 19, 20]. Gender analysis is critical when considering comorbidities between mental health and NTDs. Management of mental health and psychological stress caused by gender-related stigma as a consequence of NTDs should be integrated within health systems and services, including awareness of social stigma among health professionals [21].

## Recommendation 4: Collect and use gender-sensitive and sex-disaggregated data and implementation research to continuously improve NTD programming and ensure equity

Collecting gender-sensitive and sex-disaggregated data is possible and useful. There is need to use this data to improve the gender equity and responsiveness of NTD programs, especially at district and community levels. Gaps in disaggregated data can be addressed by adding gender equity questions to coverage-evaluation surveys and data-quality assessments [22]. Funding research bodies could better promote research that links biomedical and social determinants of health with a focus on gender and equity analysis through the instigation of key indicators that are disaggregated by gender and other social stratifiers such as age and wealth [23]. Implementation research that is gender aware and built into programs will help ensure NTD programs meet the needs of people of all genders.

## Recommendation 5: Promote intersectoral working and a person-centered approach to ensure community engagement is at the center of NTD programs

NTD programs could benefit from a health systems approach that identifies positive synergies between disease specific interventions, nontargeted health services, and other sectors [24]. An intersectoral approach is also required to address the social determinants of health—for example, in the case of schistosomiasis, repeated exposure to infected water.

Taking a person-centered approach means communities should be at the very center of NTD programs so that no sections of the population are left behind in striving towards control and elimination of NTDs. Health systems need to support community participation mechanisms that not only engage people of all genders (including women and girls) at risk or suffering from NTDs but ensure their views are listened to and applied within NTD program development and implementation. Civil society can help to ensure effectiveness and community accountability that challenges gender and other social determinants in NTD program design and implementation. NTD decision makers and program implementers should ensure that steps towards gender equity are an integral part of interventions on an ongoing and permanent basis.

## Conclusion

Gender norms, beliefs, roles, access to resources, and decision-making constitute gender power relations [3, 4]. These relations intersect with other social determinants of health, such as age, socioeconomic status and structural and environmental dimensions of daily life that govern how power is embedded within social hierarchies [23]. As such, gender inequality and inequity in relation to NTDs is predominantly socially governed and therefore actionable, and the time is now. As laid out here, collecting data on gender, developing strategies to address gender inequities within all aspects of NTD programs, and building strategic cross-sectoral partnerships will support NTD goals, the SDGs, and UHC.
